# The Profession or the Public

**Published:** 1883-05

**Authors:** 


### The Profession or the Public.

The Board of Censors of the Erie County Medical Society have undertaken, with commendable earnestness, a crusade against certain persons in this city and county, who are imposing upon the credulity of the public by practicing medicine, without either diploma or license, in violation of the laws of the State. The law of 1880 provides that it shall be a misdemeanor, punishable by fine or imprisonment, for a person to practice physic or surgery in this State without legal authority. There can be no question as to the necessity of this law in the minds of the profession, and a general regret prevails that its execution has been so long delayed. Among the people, however, especially among the uneducated, the cry of persecution is raised, and a feeling of sympathy engendered towards those who are thus hindered from carrying on their nefarious pursuits. The public, therefore, may conclude that the doctors are moving in this matter with a view to improve their own interests, without for a moment considering the dangers to which the people are exposed when pretenders, without any show of professional knowledge, are permitted to deal with the responsible work of caring for the sick.

It is one of the peculiar tendencies of our institutions that even the highest and most sacred interests must be brought within the reach of the popular grasp, and no matter how humble or how illiterate the aspirant, the position sought must not be placed beyond his earnest effort. The profession of medicine feels the influence of this American principle. The ranks have been open to every seeker after professional honor and emolument, and the standard of medical education brought down to the level of the lowliest; and even this has not been sufficient to satisfy the public taste; the bars must be let down entirely and all the cattle allowed to run at large. This picture is not overdrawn. The army of quacks and pretenders in this and other States, who live on the credulity of the people, constitutes one of those marvelous instances of long-suffering and forbearance, on the part of the public, which one would think would expend its force at the very first expression of ignorance, instead of after repeated manifestations of the most lamentable incapacity and dishonesty. The people pay the bill in loss of life, in extortionate demands, in prolonged suffering and chronic invalidism, and yet they would have this same quackery go unpunished for fear that the educated physician may reap some slight advantage through the execution of the law upon the pretenders.

If pecuniary gain be the motive power, the educated physician is the gainer by the prevalence of quackery, and the more widespread the imposition, the greater the amount that comes into his coffers. But the medical profession have always maintained the position that their highest duty consists in serving as conservators of the public health, and they have a broad field of usefulness in protecting the public against the widespread imposition which exists in every community, whereby life and health is sacrificed and suffering entailed upon the unsuspecting and the ignorant.

The law of 1880 was enacted for the benefit of the public and not for the profession. The medical profession is willing to be judged by its merits. If it does not present the best skill, where is it to be found? Do the quacks hold the panacea for ills that baffle the educated physician? Are the long course of study and the years of experience of no importance in estimating the ability of the physician, in deciding the question whether he is entitled to public confidence?

The cry of persecution with a view to excite sympathy for unworthy objects is intended for popular effect. We think the medical profession has the important duty to perform, to use its power and influence to guard life against disease, and to protect the public against those who, through ignorance, trifle with human life. We trust the censors will be encouraged to go on with the good work they have inaugurated.

